# Case Series: Removal of Rectal Foreign Bodies

**DOI:** 10.7759/cureus.13234

**Published:** 2021-02-08

**Authors:** Sharjeel Khan, Sadia Khan, Tariq Chalgari, Riaz Akhtar, Malak Asad, Besham Kumar

**Affiliations:** 1 General Surgery, Liaquat University of Medical and Health Sciences, Jamshoro, PAK; 2 Internal Medicine, Jinnah Postgraduate Medical Centre, Karachi, PAK

**Keywords:** rectum, foreign body, surgery

## Abstract

Introduction: Foreign rectal body is one of the less common presentations in the emergency department and has a variety of etiologies. Our aim is to study the mode of injury, clinical presentation, diagnosis, surgical intervention and outcomes associated with a rectal foreign body.

Methods: This cross-sectional case series was conducted from January 2019 to July 2019 in the surgical unit of a tertiary care teaching hospital in Pakistan. Mode of injury was classified as voluntary - for sexual gratification, involuntary ingestion, assault and fall. We also noted the presenting complaint, diagnosis, surgical intervention and outcome of the case.

Results: Foreign body in the rectum was more common in men (86.3%) than women (13.7%). The mean age of participants was 40 ± 15 years. Various causes include sexual gratification (45.4%), involuntary ingestion (27.2%), assault (22.7%) and history of fall (4.5%). Participants were diagnosed with sub-acute intestinal obstruction (59%), peritonitis (22.7%) and perianal injury (36.3%).

Conclusion: Sexual gratification was the most common reason for the retained rectum body. Timely diagnosis and management are required to prevent perforation and improve prognosis.

## Introduction

Colorectal foreign body insertion is not a common presentation in the emergency or surgery department. The true incidence of rectal foreign body insertion is not known [1]. Objects can be inserted in the rectum for diagnostic or therapeutic purposes, criminal assaults, drug trafficking or sexual purposes [2]. Most objects are inserted through rectum; however, some are swallowed and pass the small intestines and most of the large intestine and are stuck in the rectum [3].

The retained foreign body is diagnosed via medical history, digital rectal examination or radiographic interventions. The management requires an individual approach to injury to the rectum, size, type, shape and the time of insertion of the object [4]. Patients with the rectal foreign body present with anal or pelvic pain, anal bleeding, constipation and acute abdomen in cases with infection or perforation [5]. Cawich et al. report that the incidence of perforation is almost 10% in patients with the retained rectal foreign body [6]. Extraction of the foreign body can be done at the bedside under sedation or in the operating room under general anesthesia (GA), depending upon the severity of injury to the rectum and distal colon [6].

The aim of this case series is to identify the mode of injury, clinical presentation, diagnosis, surgical intervention and outcome associated with a rectal foreign body.

## Materials and methods

This case series was conducted in the surgical unit of a tertiary care teaching hospital in Pakistan from January to July 2019. All patients presenting with a foreign body in the rectum were included in the study after attaining informed consent. Patient anonymity was maintained while filling a questionnaire. Ethical review board approval was taken from the institute.

Patient characteristics, such as gender, age and mode of injury, were noted in the self-structured questionnaire. Mode of injury was categorized as voluntary - for sexual gratification, involuntary ingestion, assault and fall. Presenting complaint, diagnosis, surgical intervention and outcome were noted.

Statistical analysis was done using statistical package for social sciences (SPSS) version. 23.0 (IBM Corporation, Armonk, NY, USA). Continuous variables were analyzed via descriptive statistics and were presented as means and standard deviations (SDs), while categorical values were presented as frequency and percentages.

## Results

Twenty-two (22) patients presented with foreign body in the rectum during the study period. All patients were included in the case series. There were 19 (86.3%) males and three (13.7%) females. The mean age of participants was 40 ± 15 years. Mean hospital stay was 3 ± 1 days. Ten (45.4%) participants purposefully inserted foreign body for sexual gratification, six (27.2%) participants had foreign body due to involuntary ingestion, five (22.7%) participants had a foreign body due to assault and one (4.5%) participant had foreign body due to history of fall. Thirteen (59%) participants had a diagnosis of sub-acute intestinal obstruction, five (22.7%) participants had peritonitis, eight (36.3%) participants had perianal injury (Table 1).

**Table 1:** Patient demographics, mode injury and surgical intervention Abbreviations: SAIO: Sub-acute intestinal obstruction, DnE: Dilation and extraction, GA: General anesthesia, PR: Per rectal, n: number

Mode of injury	Foreign body extracted	Patient characteristics		Clinical characteristics		Surgical intervention	Outcome
		Gender	Age (in years)	Presenting complaints	Diagnosis		
For sexual gratification (n=10)	Plastic toothbrush case	Male	41	Abdominal pain	SAIO	Laparotomy	Discharge without complications
	Candle	Male	52	Abdominal pain	SAIO	Laparotomy	Discharge without complications
	Spray candle		36	Abdominal pain; constipation	SAIO; peritonitis	Laparotomy	Discharge without complications
	Batteries	Male	51	Abdominal pain	SAIO	Laparotomy	Discharge without complications
	Batteries	Male	28	Abdominal pain; constipation	SAIO	Laparotomy	Discharge without complications
	Beverage bottle	Male	56	Abdominal pain	SAIO; peritonitis	Laparotomy	Discharge without complications
	Beverage bottle	Male	21	Abdominal pain	SAIO	Laparotomy	Discharge without complications
	Beverage bottle	Male	36	Abdominal pain; constipation	SAIO; peritonitis	Laparotomy	Discharge without complications
	Beverage bottle	Male	45	Abdominal pain; constipation	SAIO; peritonitis	Laparotomy	Discharge without complications
	Beverage bottle	Male	28	Abdominal pain; constipation	SAIO; peritonitis	Laparotomy	Discharge without complications
Involuntary ingestion (n=6)	Bone	Male	31	PR bleed; abdominal pain	Perianal injury	Rectal DnE under GA	Discharge without complications
	Toothpick	Male	27	Perianal pain, peritonitis	Perianal injury	Laparotomy	Discharge without complications
	Betel nuts	Male	25	Perianal pain exacerbating during defecation	Perianal injury	Rectal DnE under GA	Discharge without complications
	Betel nuts	Female	44	Abdominal pain; constipation	SAIO	Laparotomy	Discharge without complications
	Betel nuts	Male	51	Abdominal pain; constipation	SAIO	Laparotomy	Discharge without complications
	Betel nuts	Male	39	Abdominal pain and distention	SAIO	Laparotomy	Discharge without complications
Assault (n=5)	Iron rod	Male	26	PR bleed	Perianal injury	Rectal DnE under GA	Discharge without complications
	Iron rod	Female	21	Perianal pain; PR bleed	Perianal injury	Rectal DnE under GA	Discharge without complications
	Bat handle	Male	22	Perianal pain	Perianal injury	Rectal DnE under GA	Discharge without complications
	Glass bottle	Male	29	Perianal pain; PR bleed	Perianal injury	Rectal DnE under GA	Discharge without complications
	Wooden handle	Male	36	Perianal pain; PR bleed	Perianal injury	Rectal DnE under GA	Discharge without complications
Fall (n=1)	Glass pieces	Male	25	PR bleed	Perianal injury	Rectal DnE under GA	Discharge without complications

## Discussion

Recently, cases of the foreign rectal body (FRB) are becoming more common in the surgical department. The diagnosis and management of such cases pose a challenge for the surgeon as these patients present very late and there is reluctance from patients in giving a detailed history of the incident due to stigma in our culture [7].

In this study, 22 patients were assessed in a tertiary care hospital that presented with FRB. Most of the patients were male (86.3%). In a similar study, Schellenberg observed 33 patients of FRB and found out that 85% patients were male. He also observed that most of the injuries were partial thickness and the authors demonstrated that patients with partial thickness who underwent non-operative management had a significantly lower number of stay in hospital than patients who had operation [8]. All the patients in our study, who were treated with either non-conservative method or had surgical management, were discharged without any complications. The most common reason for FRB was found to be purposefully inserted foreign body for sexual gratification (45.4%). Other causes include foreign body due to involuntary ingestion (27.2%), foreign body due to assault (22.7%) and foreign body due to the history of fall (4.5%). In a meta-analysis on FRB, sexual gratification was found to be the most common motivation, where 35.7% self-inserted sexual devices while 17.5% were found to have used glass objects [9]. The mean age of the sample size was found to be 40 ± 15 years. A 10-year long study in a single center also found out the mean age to be 38.5 ± 13.7 years [10].

Many participants in the present study had a diagnosis of sub-acute intestinal obstruction (59%), some of the participants had peritonitis (22.7%) and 36.3% of the participants had perianal injury. The most dangerous complication of FRB is perforation. These patients should be first managed as a trauma patient. And then depending on the time of presentation and type of injury, they are treated surgically either by creating a stoma or through primary repair [11]. Surgeons usually prefer laparoscopic approach, whereas in selected patients open procedures become necessary [12]. In the present study, the majority of the patients had laparotomy while the remaining patients were treated with rectal dilation and extraction (DnE) under GA.

Common signs and symptoms of patients with FRB include abdominal pain, perianal pain, per-rectal bleed and rarely constipation [3,13]. The first step of management is a physical examination to assess the signs of peritonitis and/or sepsis due to perforation [11]. Tachycardia, hypotension, severe abdominopelvic pain and fevers are indicative of a perforation. In case of a diagnosed peritonitis, early resuscitation with intravenous fluids and broad-spectrum antibiotics become the mainstay of treatment. Rectal foreign bodies can also be palpated transabdominally, mostly in left quadrant. Rectal examination can also reveal foreign objects, which is done only after abdominal X-ray has excluded the presence of any sharp object that can injure the doctor [14].

In a stable patient, X-ray and/or CT scan can be performed to further determine the size, shape and position of FRB, and to evaluate the presence of pneumoperitoneum. Depending on the size and shape, the FRB can be removed in an emergency room via a trans-anal approach or in an operating theater when an abdominal approach is mandated [3,14,15].

Flexible proctoscopy and X-ray films are encouraged to obtain after removal in order to evaluate the status of the rectum and rule out ischemia or wall perforation. Post removal observation is mandatory and depends on the clinical status of the patient, whether there was a delay in the presentation or not, the presence of any comorbidities and any complication of the treatment [3,13].

It is important that postoperative management should also include counselling of patients, particularly who had retained foreign body as a result of sexual gratification.

## Conclusions

FRB is becoming increasingly common in our society and still poses a difficult diagnostic and management dilemma. Most of the patients are males and a majority of them belong to the middle-age group. Sexual gratification is one of the main reasons of FRB in both developing and developed countries. Diagnosis is made on a detailed history, physical examination and radiological evaluation. Patients are divided into two categories; stable and unstable on this basis. Stable patients are further managed via open or laparoscopic approach depending on the position, size and shape of FRB. Unstable patients are treated according to advanced trauma life support (ATLS) protocol and then managed accordingly. Post removal observation is mandatory and evaluation of any injury due to surgery via X-ray or proctoscopy is encouraged.
